# Draining phenomenon in closed narrow tubes pierced at the top: an experimental and theoretical analysis

**DOI:** 10.1038/s41598-018-32359-5

**Published:** 2018-09-20

**Authors:** Amit Kumar, Subhabrata Ray, Gargi Das

**Affiliations:** Department of Chemical Engineering, Indian Institute of Technology Kharagpur, West Bengal, 721302 India

## Abstract

The phenomenon of draining, although ubiquitous in nature, has received scant attention especially in the meso-scale. We observe that closed top tubes drain by the inception of an axisymmetric ‘Taylor finger’ while a minute pierce of the top closure results in an altogether different physics with air entry from the top pushing the liquid out. Again, a coupled mechanism comprising full bore followed by film draining is observed for “too small” a top pierce at “high enough” Eotvos number. Top pierce initiates draining in dimensions which would not drain otherwise and finger entry hastens the process of draining. The myriad of phenomena thus exhibited is depicted as phase diagrams in vertical and inclined conduits. A mechanistic model has been proposed to predict draining and the onset of finger entry in vertical tubes.

## Introduction

It is common knowledge that emptying of a vertical cylinder open to atmosphere corresponds to Galilei’s problem of solid free fall where the velocity of the liquid surface increases with progress of draining. On the other hand, the phenomenon obeys Torricelli’s law when the same cylinder is drained through an orifice much smaller than the tube diameter^1^. For long narrow tubes closed at the top, draining occurs by the admission of a single elongated air finger from the open end. The finger grows and fills the entire tube while liquid drains as an annular film through the space between the growing finger and the conduit wall^2,3^. The phenomenon is continuous and the physics of draining is governed by finger dynamics^2^. For the same closed top conduit emptying through an opening much smaller than the conduit diameter, the draining pattern is oscillatory^4^ and characterized by two distinct time scales, the long time scale of emptying and the short time scale of interface oscillation.

In this article, we discuss the phenomenon of draining from the open end of conduits when their top closure is pierced by small amounts, a geometry hitherto unexplored, and compare the behavior with emptying of closed top and open top tubes. We confine our studies to the meso-scale^5^ where gravity and surface forces compete to govern the hydrodynamics of flow. Experiments performed over a wide range of conduit diameter, inclination and liquid surface tension reveals several unique features of the draining process. The results are summarized as phase diagrams for vertical and inclined tubes. A mechanistic analysis validated with experiments is proposed to predict the rate and time of draining in vertical tubes. The physics thus unraveled is expected to facilitate draining in the non-viscous surface tension dominant domain, an upcoming challenge in the current trend of miniaturization. The non-viscous meso-scale domain is often encountered in monolith reactors, process and food industries, biomedical applications and surgical drains where an accurate estimate of drainage output can identify any complications resulting in fluid leakage and can also assist in designing systems for intravenous replacements of fluids.

## Experimentation

**Figure 1:**
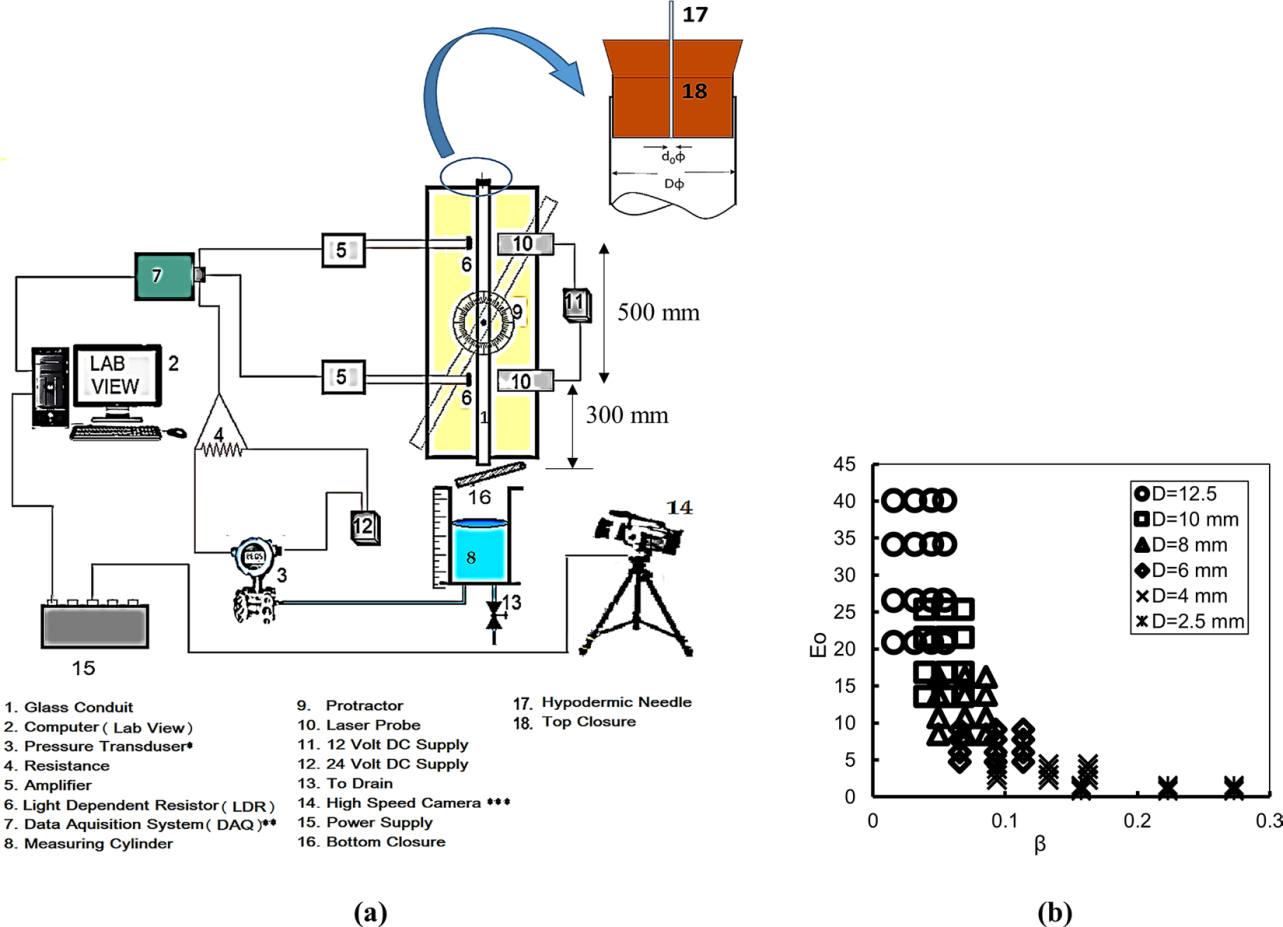
(**a)** Schematic of experimental arrangement. *Honeywell, model 120 STD, full-scale accuracy of ±0.1%, least count of 1.0 × 10^−2^ Pa and ±2% uncertainty, range 4–20 mA. **National Instruments, USA, model no-USB-6218. ***Vision Research, USA Phantom MICRO LC-320. (**b)** Investigation domain in Eo - β plane. Each Eo- β combination has been investigated at conduit inclinations of 15°, 30°, 45°, 60°, 75° and 90° with respect to horizontal.

The experimental facility (Fig. 1(a)) comprises of a bank of glass tubes, initially full of liquid. The geometrical parameters (D, θ) are changed using six different tubes of 2.5, 4.2, 6, 8, 10 and 12.5 mm diameter, each of 1.2 m length and experiments are conducted at six different inclinations (15°, 30°, 45°, 60°, 75° and 90° with horizontal) for each tube. The inner diameters (D) are noted by measuring the volume of water required to fill known lengths of the tubes and verified from measurements by a vernier caliper. The angle of inclination with respect to the horizontal (θ) is measured by a protractor (9) fitted at the center of each tube, with its fulcrum for rotation in a vertical plane.

The physical properties of the fluids are varied by adding surfactant (SDS) at different concentration 1000 ppmw, 2000 ppmw and 3000 ppmw) to water. The surfactant changes the surface tension of water while its density and viscosity remains the same (ρ = 998 kg/m^3^, μ = 8.9 × 10^−4^ Pa). Since we wanted to stay in the non-viscous domain, the liquids used in the experiments present a small viscosity $(400\ge \frac{g{\rho }^{2}{D}^{4}}{{\mu }^{2}L}\ge 2.5\times {10}^{5})$^4^ and differ mainly through their surface tension.

The tube and fluid selection enables an independent variation of diameter and surface tension for a wide range of Eotvos number (0.8 ≤ Eo ≤ 40) defined as the ratio of buoyancy to surface force $(Eo=\frac{{\rm{\Delta }}\rho g{D}^{2}}{\sigma })$. It is ensured that the combination of diameter range (2.5 to 12.5 mm) and surface tension (σ = 73.8 dyne/cm, 58 dyne/cm, 45.2 dyne/cm and 38.5 dyne/cm) encompasses the surface tension dominant regime $(1.9a < D < 4.5a,\,a=\sqrt{\frac{2\sigma }{\rho g}})$ proposed by Zukoski^6^ which for the present experiments is 5.3 mm < *D* < 7.3 mm.

Experiments are performed with the top closure pierced by 0, 1, 2, and 3 hypodermic needles. Care is taken to ensure that the needles (diameter 0.394 mm and length 38 mm) just pierce the cork but do not extend into the liquid since in the latter case some liquid may rise through the needle and influence the draining process.

The domain of investigation is depicted in Fig. 1(b) in (*β* − Eo) plane where *β* = *d*_*e*_/*D*, *d*_*e*_ the area equivalent diameter of top opening is ${d}_{o}\sqrt{n}$ (n = no of needles and *d*_*o*_ = needle inside diameter).

Before each individual run, the tubes are cleaned thoroughly with detergent solution, rinsed with water and methanol and allowed to dry in order to ensure that the capillary wall characteristics are the same for each experiment. They are then filled with liquid and oriented at a particular inclination. The closure (16) at the bottom of the tube is removed quickly and the draining liquid is collected in a measuring cylinder (8) placed on the ground at a distance of about 1 m from the open end of the emptying capillary. This ensures that the liquid falls freely out of the tube and avoids the influence of the limit condition (at x = −1 m) on the interface trajectory x (t). The measuring cylinder contains a known volume of liquid prior to draining in order to minimize errors due to splashing of the draining liquid.

The volume drained as a function of time and the time of draining are estimated from high speed videographic recording (500 fps) of the liquid surface in the measuring cylinder. The data thus obtained is further validated from the signals of a pressure transducer connected to the bottom of the measuring cylinder. The transducer (details in Fig. 1) is activated prior to the draining process and the signals recorded in a PC via a DAQ (7) gives the cumulative volume drained as a function of time. The two methods agree within a few percent. Each experiment has been repeated at least five times and the uncertainty of measurement is obtained as ±0.88%.

## Typical Draining Curves

**Figure 2:**
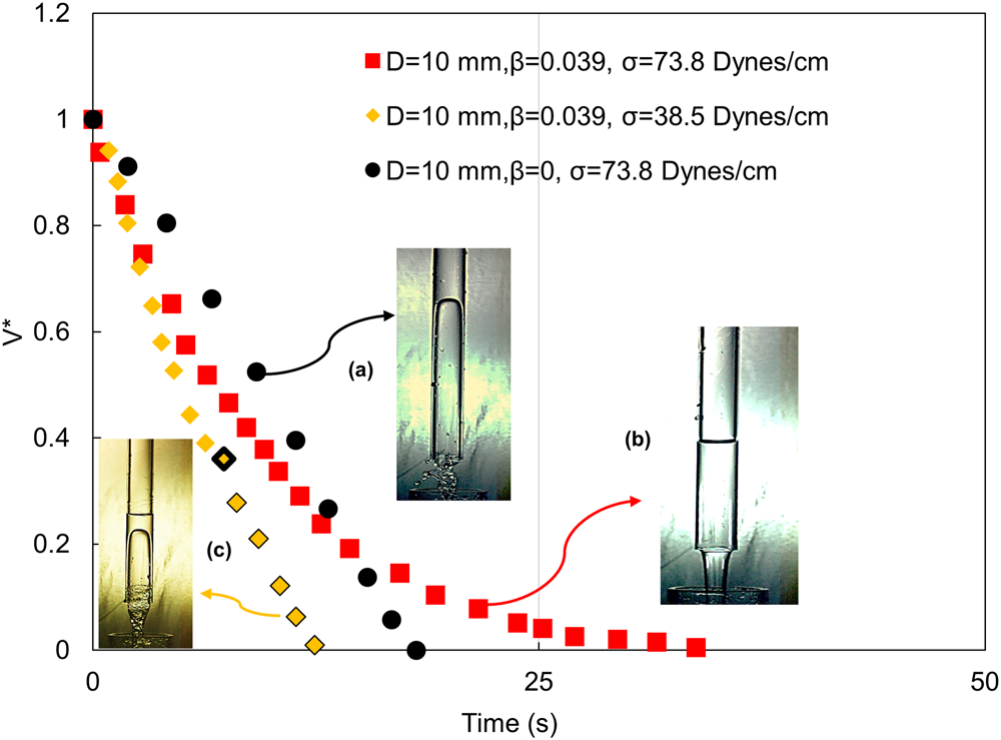
Typical draining curves obtained in the present study. (**a**) Film draining due to Taylor finger growth in closed top tubes. (**b**) Full bore draining due to air entry from top in pierced top tubes. (**c**) Coupled mechanism (full bore followed by film draining) for “small” top pierce (**a**) is denoted by black, (**b**) by red and (**c**) by yellow legends throughout the paper. Black outline in yellow legends signify admission of Taylor finger.

The experiments have revealed three types of draining curves as depicted in Fig. 2. These are presented in non-dimensional co-ordinates as the fraction of conduit volume retained, V* ( = *(V*_*T*_ − *V(t))/V*_*T*,_ where *V*_*T*_ = total liquid volume filling the tube and *V(t*) = volume of the liquid drained in time t) versus T*, the elapsed time, scaled by the characteristic time $\sqrt{\frac{L}{2g}}$. Such a scaling eliminates the effect of conduit length on time of draining.

For closed top draining (marked by black legend), the interface recedes at a quasi-constant speed. This is expected as closed top tubes empty by the well-known mechanism of Taylor finger growth which is similar to the physics of Taylor bubble rise in liquid filled conduits and Taylor bubble rises at a constant speed in a particular conduit diameter^2,6^. No draining occurs from closed top tubes below *D* = 6 mm. This arises because the velocity of finger growth is proportional to $\sqrt{gD}$ for buoyancy dominated cases and for reduced dimensions where surface tension becomes important (1.9*a* < *D* < 4.5*a*), the velocity reduces faster than $\sqrt{D}$ till draining ceases for *D* ≤ 1.9*a*^6^ (5.3 mm–7.3 mm in the present study).

Interestingly, draining can be initiated for *D* < 1.9*a* by a mere pierce of the top closure. The physics of draining changes completely since air entering through the pierce pushes the interface down. This is denoted by a draining curve, convex towards the origin (red legend in Fig. 2), indicating a faster initial rate during full bore draining which gives way to dropwise draining as the tube almost empties. Curve (b) in Fig. 2 shows that that 90% of the tube empties within 19 sec by full bore draining while the remaining 10% requires 17 sec to drain. This can be attributed to surface tension effect that is more pronounced for smaller conduit diameters and liquids with higher surface tension. As a result, liquids usually do not drain completely in pierced top tubes and the proportion of retained liquid is higher for lower tube diameters and higher liquid surface tension.

Interestingly, below a critical relative diameter of the top pierce, draining occurs by the coupled mechanism of closed top and pierced top draining. The liquid column initially recedes due to the pressure of air entering from the top and after a critical volume of drainage, air enters from the open end as an axisymmetric bullet shaped Taylor finger. Curve (c) in Fig. 2, denoted by yellow legends, depicts the profile for coupled mechanism draining. Markers in black outline denote the onset of finger entry. The inception of Taylor finger momentarily arrests the recession of the top interface and thereafter, draining is governed by finger dynamics rather than air entering from top. As a result, the draining curve becomes linear again and the tubes drain at a faster rate. The admission of Taylor finger also ensures complete emptying of the conduits.

## Influence of Input Parameters

**Figure 3:**
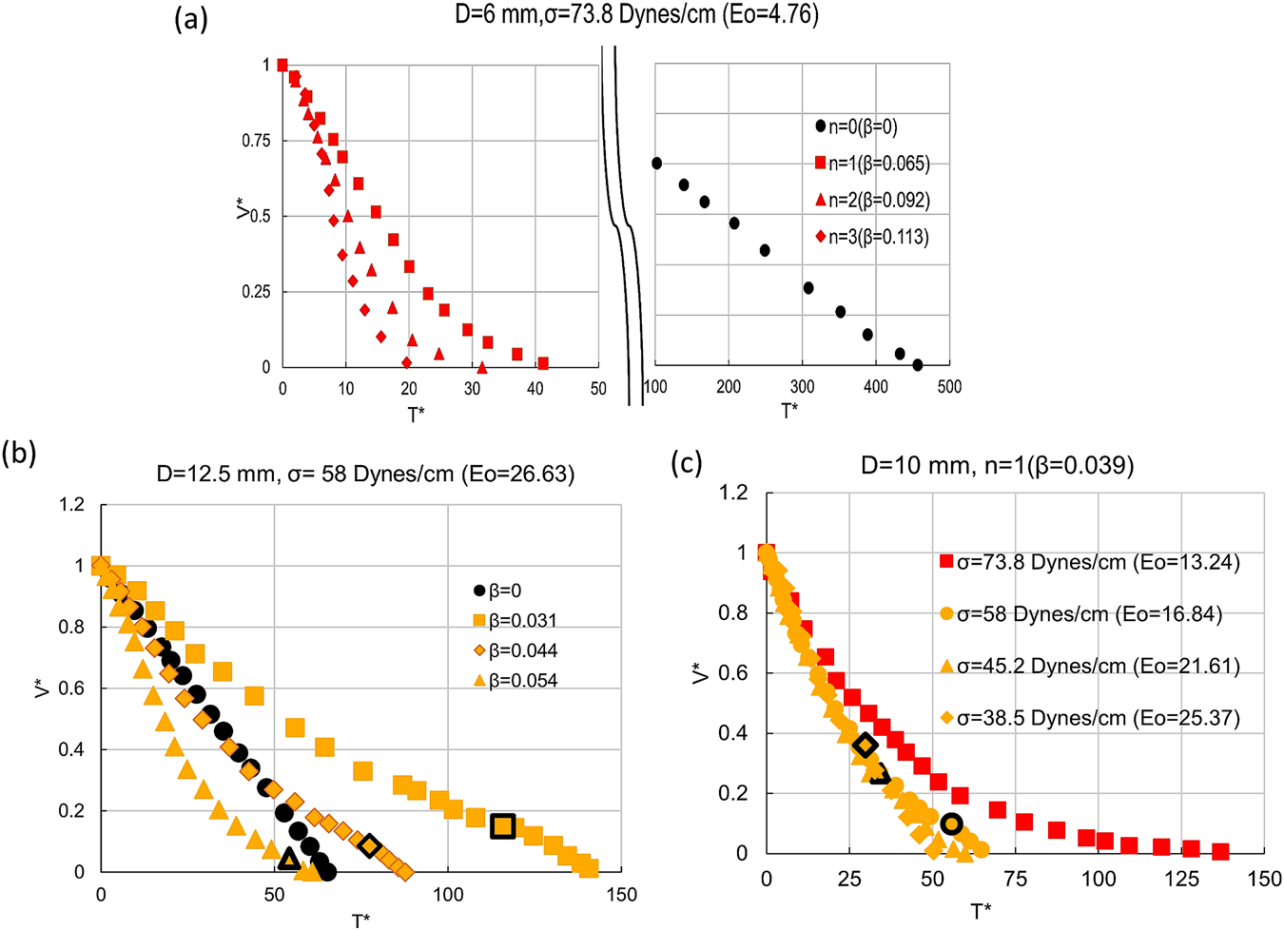
Effect of β on draining for **(a)** Eo = 4.76 (D = 6 mm, σ = 73.8 dynes/cm), **(b)** Eo = 26.63 (D = 12.5 mm, σ = 58 dynes/cm). (**c)** Effect of liquid surface tension on draining profile in 10 mm diameter tube for β = 0.039.

**Figure 4:**
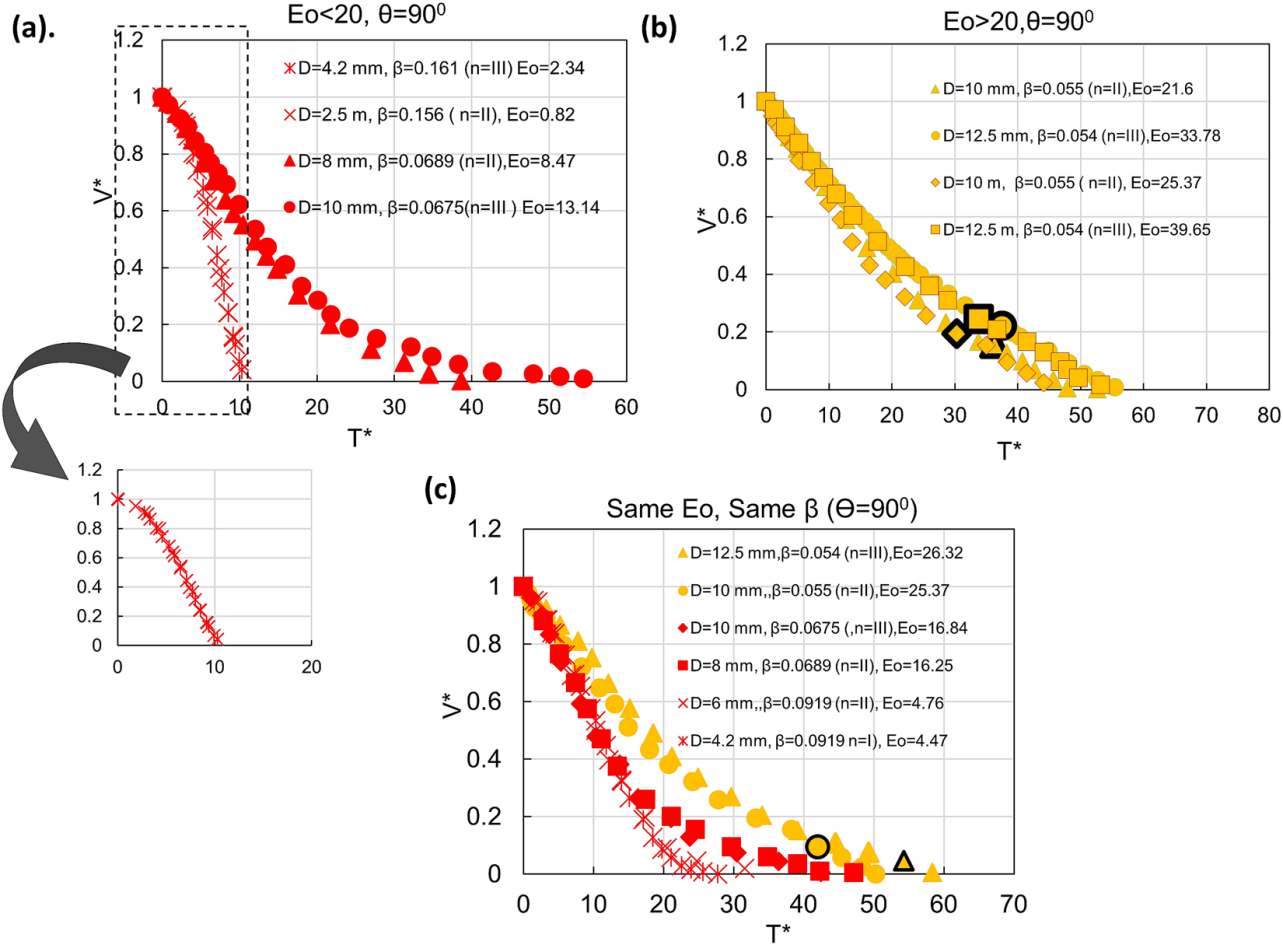
Effect of tube diameter and β on fractional volume of draining for (**a**) Eo < 20 (**b**) Eo > 20. Data points with similar β but different Eo aim to illustrate the individual effects. **(c)** Draining profiles for similar β and Eo for different conduit diameter and liquid surface tension.

To understand the physical law governing the draining process, Figs 3 and 4 presents the trajectories obtained from videographic measurements for different fraction of top opening (*β* = *de*/*D*), tube diameter (D) and surface tension (σ). The normalised volume (V*) and time axis (T*) facilitate a comparative study. For ease of understanding, the legends defined in Fig. 2 are adopted in all figures, i.e. film draining, full bore draining and coupled mechanism draining are depicted by black, red and yellow legends and Taylor finger entry in pierced top tubes is indicated by black marker on the yellow legend.

A comparison of Fig. 3(a,b) reveals that the velocity of draining for *β* = 0 is an order of magnitude higher in 12.5 mm as compared to 6 mm conduit. Apart from the differences in the shape of the trajectory in closed top and pierced top cases, the emptying time (*T*_*D*_) is an order of magnitude larger for β = 0 as compared to β ≠ 0 in the 6 mm diameter conduit (Fig. 2(b)) while in 12.5 mm conduit, the time of draining (*T*_*D*_) is lower for β = 0 as compared to β < 0.05 (n = 1 and n = 2). As expected, the draining rate in pierced top conduits increases with fractional opening (Fig. 3(a)) since the pressure above the liquid column (*P*_*i*_) increases with β. Interestingly, for larger tube diameters (Fig. 3(b)), a decrease in β increases the propensity of finger entry. From Fig. 3(c) we observe that a decrease in σ hastens draining by a twofold effect of decreasing surface tension and increasing propensity of finger entry.

Figure 4(a) demonstrates that the top pierce can enable draining from small enough tubes where capillary effects prevent finger formation. A close observation of the inset reveals S – shaped trajectories for *β* ≥ 0.093 and Eo < 20 (for 4.2 and 2.5 mm tubes). Clanet (2000)^1^ had reported similar curves during draining of closed top tubes through an orifice for orifice/conduit diameter >0.0625 and suggested that these display a transition to the Galilean regime. The antagonistic role of surface tension and conduit diameter is observed in all the experiments, thus highlighting the importance of Eotvos number in the meso-scale.

## Effect of Eotvos number and **β**

In order to pinpoint the effect of Eo and *β*, draining trajectories are presented for same Eo different *β* and (nearly) same β and different Eo in Fig. 4. We observe a pronounced effect of β irrespective of Eotvos number for Eo ≤ 20 (Fig. 4(a)) while for Eo > 20, (Fig. 4(b)) the effect of Eo (conduit diameter in particular) seems to be more prominent and for same *β*, draining is faster in the larger diameters.

Figure 4(c) further shows that data points for same *β* and Eo over a wide range of input variables merge in a single curve which confirm these to be the dominant parameters influencing draining in the mesoscale, the only difference arising after inception of Taylor finger.

**Figure 5:**
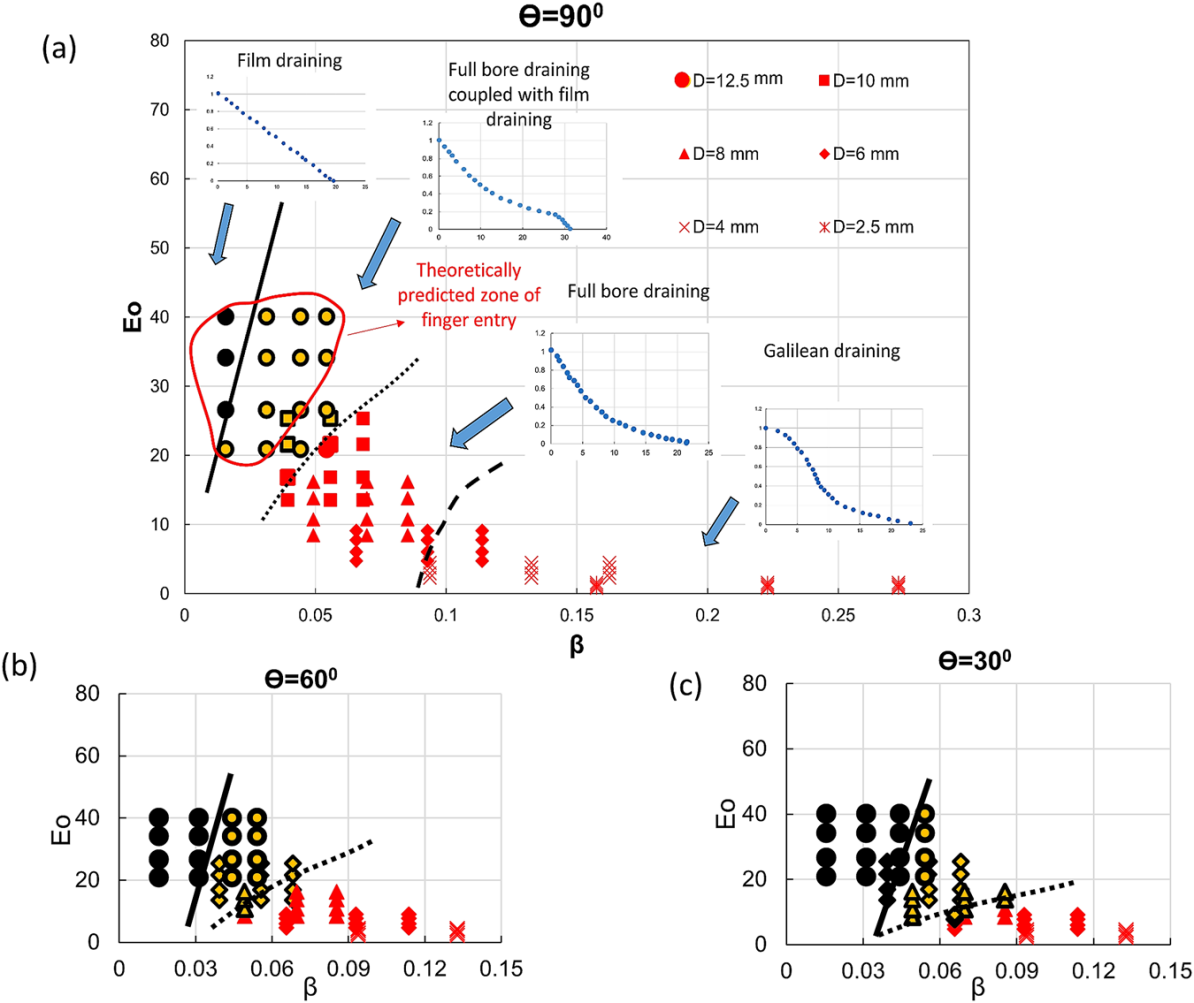
Phase diagram for draining in the meso scale (**a**) θ = 90° (**b**) θ = 60° (**c**) θ = 30^0^. Zone of finger entry as predicted from theory in vertical tubes is shown by the red-bordered region in (**a**).

This suggests that the different draining phenomena can be summarized as a phase diagram with Eo and *β* as axes. The map presented in Fig. 5(a) demarcates regimes of film draining and full bore draining with a transition zone between the two. The onset of Galilean draining characterized by S- shape trajectories at higher values of *β* and low Eo is also marked in the figure. We note that a lower surface tension and higher conduit diameter (higher Eo) facilitates finger formation for the same β. This is because with increase in Eo, the Taylor finger requires lower energy to rupture the liquid interface and rise. It is interesting to note that finger entry occurs only for conduit diameter where finger entry can hasten the process of draining i.e. when draining is faster for n = 0 as compared to n ≠ 0. Or else the entire draining process is governed by air pressure from the top.

## Mathematical Modeling

Based on experimental observations, we propose a mechanistic analysis to predict draining from vertical conduits of diameter *D* and length *L*, partially closed at top and open at the bottom. Closed and open top draining are the limiting cases of the phenomena. The conduit, initially filled with a liquid of density ρ, viscosity μ, surface tension σ and contact angle *α* with the conduit wall, starts draining at t = 0 when the bottom cover is opened.

**Figure 6:**
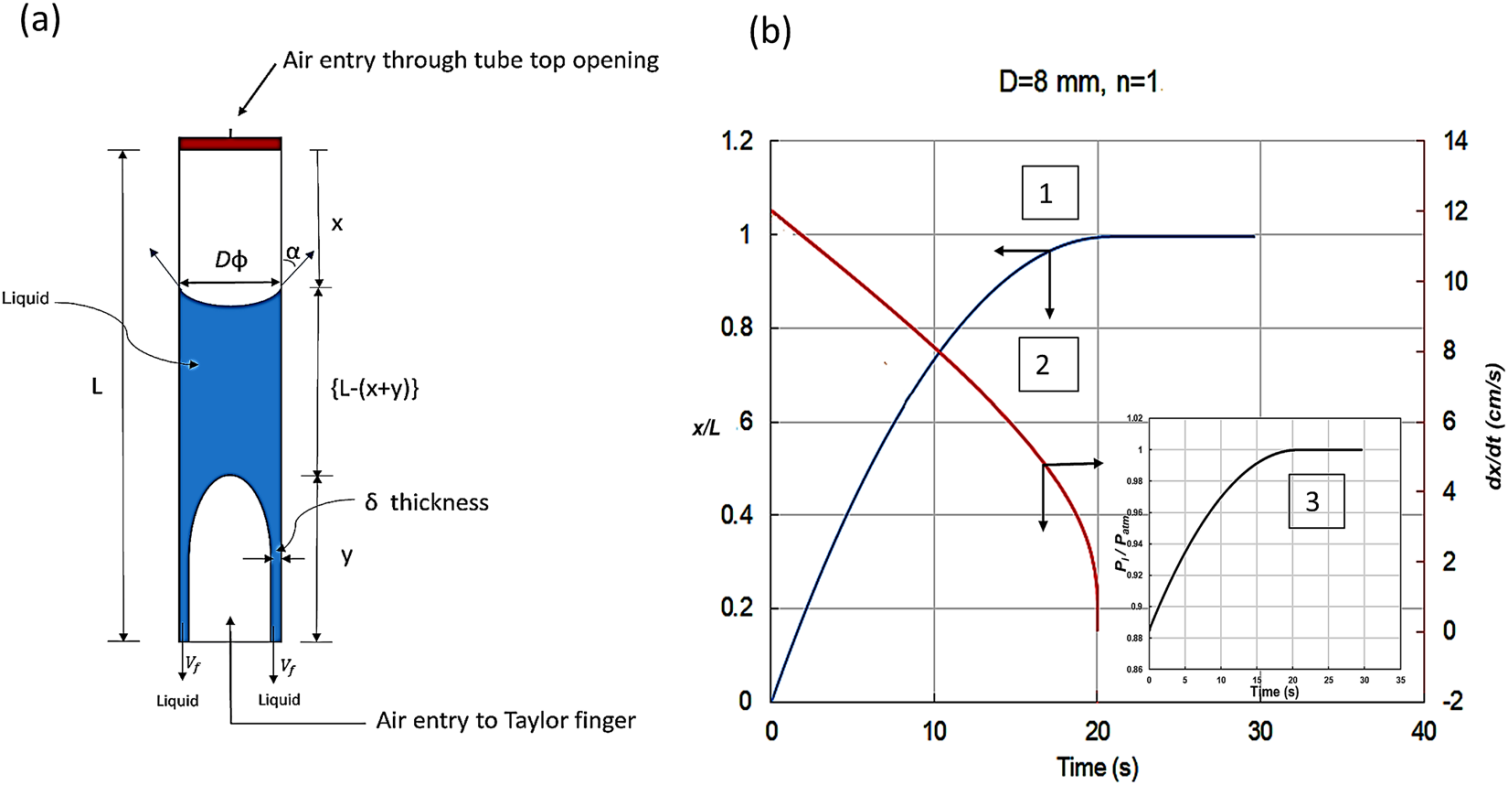
(**a**) Schematic showing nomenclature used in the model for vertical tubes (**b**) Theoretical prediction of (1) draining trajectory, (2) rate of draining (*dx*/*dt*) and (3) *in situ* pressure above the liquid column as a function of time in 8 mm diameter conduit for β = 0.048 (n = 1).

We formulate the time (*T*_*D*_) and rate $(\frac{dV}{dt})$ of draining from narrow conduits and also propose the condition for finger entry. Considering that for 0 < *β* < 1, initially full bore draining occurs through a distance *x* from the top (with air entering through the pierce) and after a point, Taylor finger forms and gives rise to film draining as an annular ring of thickness, *δ*. The finger grows for a distance *y* till it meets the air-liquid interface and draining is complete [See Fig. 6(a)].

The forces acting on the descending liquid mass are surface tension (*F*_*s*_) and frictional force (*F*_*f*_) opposing draining and gravity (*F*_*g*_) favoring the recession process

1 $${F}_{s}=\pi D\sigma \,\cos \,\alpha $$

2 $${F}_{f}={f}_{1}\frac{(L-x){\rho }_{w}{(\frac{dx}{dt})}^{2}}{2D}\frac{\pi {D}^{2}}{4}+{f}_{2}\frac{y{\rho }_{w}{{V}_{f}}^{2}}{2D}\frac{\pi {D}^{2}}{4}$$

3 $${F}_{g}={\rho }_{w}\,g\{(L-\overline{x+y})\frac{\pi {D}^{2}}{4}+\pi \delta (D-\delta )y\}$$

In Eqs (1–3) *V*_*f*_ is the average velocity of draining after finger entry, *α* is the contact angle and *f*_1_ and *f*_1_, are Darcy’s friction factor expressed as

$$f=64/Re\,for\,Re\le 4000$$

and

4 $$\frac{1}{\sqrt{f}}=-\,2\,{\mathrm{log}}_{10}(\frac{\varepsilon }{3.7D}+\frac{2.51}{{\rm{Re}}\sqrt{f}})\,\,{\rm{for}}\,{\rm{Re}} > 4000\,[{\rm{Colebrook}}\,{\rm{equation}}]$$

*ε*, the surface roughness = 0 for smooth glass^7^ tubes and

$$Re=D(dx/dt)\rho /\mu \,{\rm{f}}{\rm{o}}{\rm{r}}\,{\rm{f}}{\rm{u}}{\rm{l}}{\rm{l}}\,{\rm{b}}{\rm{o}}{\rm{r}}{\rm{e}}\,{\rm{d}}{\rm{r}}{\rm{a}}{\rm{i}}{\rm{n}}{\rm{i}}{\rm{n}}{\rm{g}}$$

and

5 $$Re=\delta {V}_{f}{\rho }_{w}/{\mu }_{w}\,{\rm{for}}\,{\rm{film}}\,{\rm{draining}}$$

where, *δ* is annular film thickness

The resulting rate of change of liquid momentum is

6 $$\frac{d(mv)}{dt}=\frac{d}{dt}[\frac{\pi {D}^{2}}{4}\{L-(x+y)\}{\rho }_{w}\frac{dx}{dt}]+\frac{d}{dt}({\rho }_{w}\delta \pi Dy{V}_{f})$$

where the mass flow rate of inflowing air through the pierced top considering adiabatic flow^8^ is -

7 $$\dot{m}={C}_{D}{A}_{o}{\rho }_{atm}\sqrt{{P}_{atm}/{\rho }_{atm}}\sqrt{\frac{2\gamma }{\gamma -1}[{(\frac{{P}_{i}}{{P}_{atm}})}^{\frac{2}{\gamma }}-{(\frac{{P}_{i}}{{P}_{atm}})}^{\frac{\gamma +1}{\gamma }}]}$$

*A*_*o*_ is the opening area of top pierce and *C*_*D*_ is its discharge coefficient. Since the opening is quite small, we assume *C*_*D*_ to be a function of *β* only, independent of Reynolds number^9^ of air and propose the following expression obtained from experimental data -

8 $${C}_{D}=0.0732+\frac{0.4421}{\{1+{e}^{(6.1029-49.02\beta )}\}}$$

Since draining is a result of the combined effect of falling interface with velocity $\frac{dx}{dt}$ and Taylor finger growth with velocity *k*,

9 $${V}_{f}=\frac{dx}{dt}+\frac{k}{4}\frac{{(D-2\delta )}^{2}}{\delta (D-\delta )}$$

Assuming air above the liquid column to be an ideal gas of density *ρ*_*i*_

10 $$\frac{dx}{dt}=\frac{\dot{m}}{{\rho }_{i}\pi {D}^{2}/4}-\frac{0.351x}{{\rho }_{i}{P}_{atm}(273+{T}_{atm})}\frac{d{P}_{i}}{dt}$$

Since our experiments show that Taylor finger exhibits the same characteristics during growth in closed top and pierced top tubes, the value of *k* measured in closed top tubes by the optical probe technique^10^ is used in Eq. (9). The technique estimates *k* within ±1.5% from signals recorded by a pair of probes located 500 mm apart as shown in Fig. 1. We observe that the velocity remains constant and can be correlated satisfactorily with the correlation proposed by Wallis^11^ for rise of Taylor bubbles in stagnant liquids.

11 $$k=0.345\{1-{e}^{(\frac{3.37-Eo}{10})}\}\sqrt{\frac{\{gD({\rho }_{w}-{\rho }_{g})\}}{{\rho }_{w}}}$$

Substituting the value of *V*_*f*_ and *k* from Eqs 9 and 11 in Eq. 6 and equating the rate of momentum change to the net impressed force gives -

12 $$\begin{array}{l}\frac{\pi {D}^{2}{\rho }_{w}}{4}[\{L-(x+y)\}\frac{{d}^{2}x}{d{t}^{2}}-k(\frac{dx}{dt})-{(\frac{dx}{dt})}^{2}]\\ \,\,+\,\pi D\delta {\rho }_{w}\{k(\frac{dx}{dt})+({k}^{2}/4)\frac{(D-2\delta )}{\delta (D-\delta )}+y\frac{{d}^{2}x}{d{t}^{2}}\}\\ \begin{array}{rcl} & = & \frac{\pi {D}^{2}{\rho }_{w}}{4}\{L-(x+y)\}g+\pi D\delta {\rho }_{w}yg\\  &  & -\,\,{f}_{1}\frac{\{L-(x+y)\}{(\frac{dx}{dt})}^{2}}{2D}-{f}_{2}\frac{y{{V}_{f}}^{2}}{2D}+\frac{\pi {D}^{2}}{4}{P}_{i}-\frac{\pi {(D-2\delta )}^{2}}{4}{P}_{atm}\end{array}\end{array}$$

with the initial condition (at t = 0)

$$x=0,\,dx/dt=0;{P}_{i}={P}_{atm}-\rho gL$$

The only unknown in Eq. (12) is *δ*. We use measurements of rate of liquid draining (*dQ*_*L*_/*dt*) and finger growth velocity (*k*) to obtain an estimate of normalized film thickness δ* (=*δ*/*D*). Assuming Taylor finger to be incompressible during growth, δ* is obtained as-

13 $$\delta \ast =\frac{1}{2}(1-\sqrt{\frac{4(d{Q}_{L}/dt)}{\pi {D}^{2}k}})$$

Following the cue from literature^12–14^, we correlate our experimental data to obtain an empirical equation for δ* in terms of Capillary number $(\frac{\mu k}{\sigma })$, the ratio of viscous to surface forces.

14 $${\delta }^{\ast }=(\delta /D)=0.16(1-{e}^{[-1000(\frac{\mu k}{\sigma })]})$$

Substituting Eq. (14) in Eq. (12) provides the desired solution

### Condition of finger Entry

We postulate that Taylor finger enters pierced top conduits when *P*_*atm*_ exceeds the summation of pressure forces due to liquid hydrostatic head, its kinetic head due to velocity of downflow, pressure (*P*_*i*_) above the liquid and the surface tension force necessary to rupture the liquid surface for finger to enter. Mathematically this gives,

15a $${P}_{i}+({\rm{L}}-{x}_{c}){\rho }_{w}g+\frac{1}{2}{\rho }_{w}{(dx/dt)}^{2}-\frac{4\sigma \,\cos \,\alpha }{{\rm{D}}} < {P}_{atm}$$

and Taylor finger does not enter i.e. *y* = *0, k* = 0 when

15b $$(L-x)+\frac{\frac{1}{2}{(\frac{dx}{dt})}^{2}}{g}-\frac{4\sigma \,\cos \,\alpha }{D{\rho }_{w}\,g} > \frac{({P}_{atm}-{P}_{i})}{{\rho }_{w}\,g}$$

Eq. (15b) thus, provides an expression to predict the relative interface location (*x*_*c*_/*L*) at the inception of finger entry in non- dimensional coordinates.

16 $$(1-{x}_{c}/L)+\frac{1}{2}(\frac{D}{L})F{r}^{2}-\frac{4F{r}^{2}}{We}(\frac{D}{L})cos\alpha =\frac{({P}_{atm}-{P}_{i})}{{\rho }_{w}\,gL}$$

implying

17 $${x}_{c}/L=f(D/L,Fr,We,{P}_{i}),\,{\rm{i}}\,.\,{\rm{e}}.\,\,{x}_{c}/L=f(D/L,Fr,We,\beta )$$

for the non-dimensional numbers defined as

$$Fr=\sqrt{\frac{{(dx/dt)}^{2}}{gD}}\,{\rm{and}}\,\,We=\frac{{\rho }_{w}D{(dx/dt)}^{2}}{\sigma }$$

Eqs (15 and 16) assumes that the pressure *P*_*i*_ at the air-liquid interface is same as the pressure in the tube above the interface, this pressure being lower than *P*_*atm*_.

The solution of the model infers the velocity of top interface (*dx*/*dt*), draining trajectory *x*(*t*) and *in-situ* pressure (*P*_*i*_) above the liquid column with time. A typical result for D = 8 mm and β = 0.048 (n = 1) is presented in Fig. 6(b). The figure shows the location of top interface (*x*) and interface receding rate (*dx*/*dt*) as a function of time (*t*). We note that the pressure above the liquid surface increases with time which is the inverse of hydrostatic pressure of liquid column. As the liquid level falls, hydrostatic pressure decreases.

## Model Validation

We solve Eqs (7, 10 and 12) with boundary conditions given by Eq. (16) to obtain (*x*_*c*_/*L*) and note that Taylor finger forms only for 10 mm and 12.5 mm conduit. The same is also evident from the experimental regime map presented in Fig. 5(a). The zone of finger entry as predicted from theory is superimposed in the regime map and shows fair agreement. Table 1 also compares the inception of Taylor finger as predicted from experiment and theory. We observe only a few points of mismatch encircled in the Table and also note that the mismatch arises when finger inception occurs nearing the end of the draining process.

**Table 1:** Taylor finger entry in pierced top tubes as predicted from theory and experiments.

Diameter (D) (mm)	No. of needles	β	Surface tension (σ) (Dynes/cm)	Eotvos Number (Eo)	Taylor finger formation (Y/N)	
					Theory	Experiments
12.5	1	0.031	72	21.45	Y	Y
12.5	2	0.044	72	21.45	**Y**	**N**
12.5	3	0.054	72	21.45	N	N
12.5	1	0.031	58	26.63	Y	Y
12.5	2	0.044	58	26.63	Y	Y
12.5	3	0.054	58	26.63	Y	Y
12.5	1	0.031	45.2	34.17	Y	Y
12.5	2	0.044	45.2	34.17	Y	Y
12.5	3	0.054	45.2	34.17	Y	Y
12.5	1	0.031	38.5	40.12	Y	Y
12.5	2	0.044	38.5	40.12	Y	Y
12.5	3	0.054	38.5	40.12	Y	Y
10	1	0.039	72	13.55	**Y**	**N**
10	2	0.056	72	13.55	**Y**	**N**
10	3	0.068	72	13.55	**Y**	**N**
10	1	0.039	58	16.83	Y	Y
10	2	0.056	58	16.83	**Y**	**N**
10	3	0.068	58	16.83	**Y**	**N**
10	1	0.039	45.2	21.6	Y	Y
10	2	0.056	45.2	21.6	Y	Y
10	3	0.068	45.2	21.6	Y	Y
10	1	0.039	38.5	25.35	Y	Y
10	2	0.056	38.5	25.35	Y	Y
10	3	0.068	38.5	25.35	N	N

**Figure 7:**
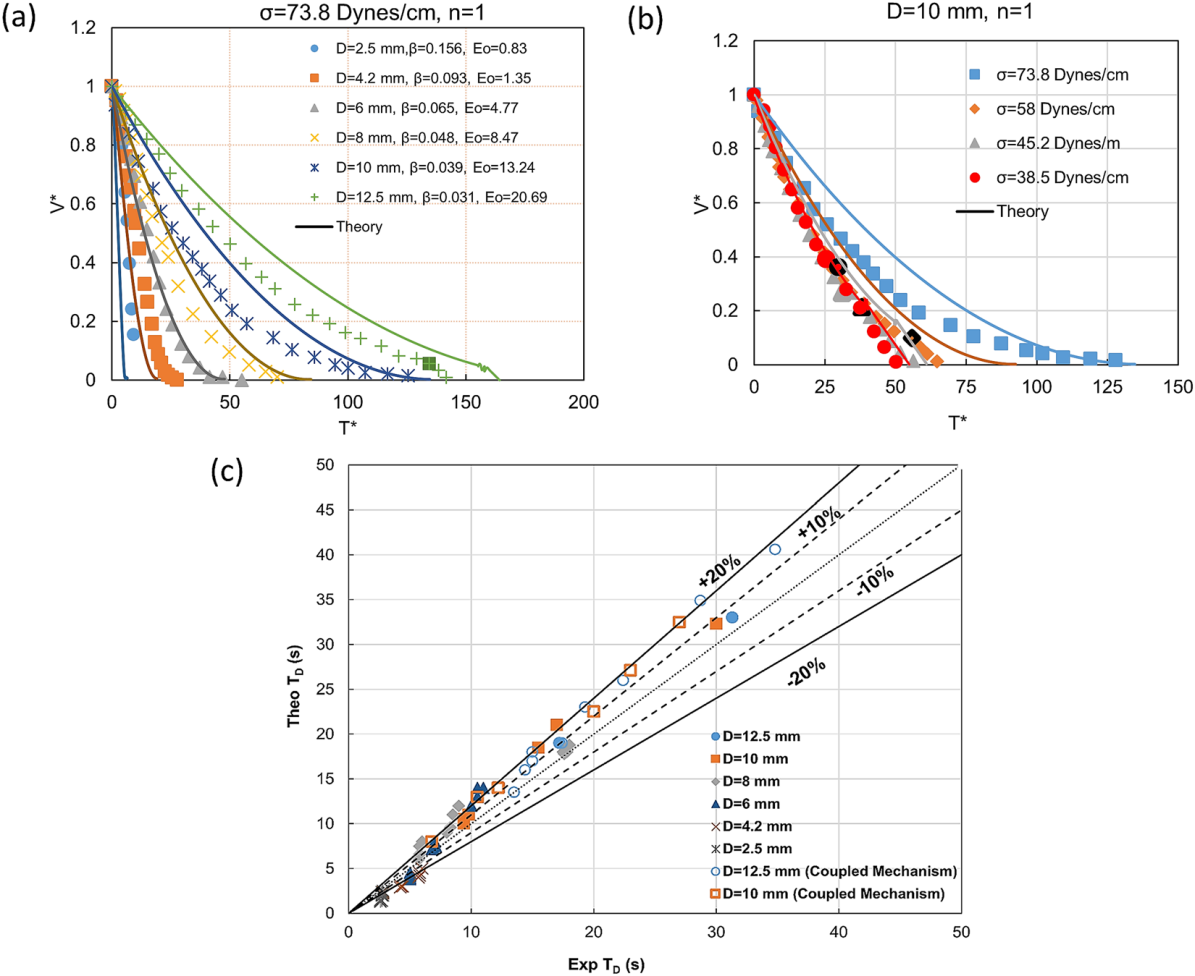
Validation of model proposed for vertical tubes. Comparison of (**a**) draining profile for water in different tube diameters at n = 1 (**b**) draining profile with liquid surface tension (σ) as parameter in D = 10 mm and n = 1. (**c**) Total time of draining (T_D_) as predicted from theory and experiments for full bore (D = 2.5–8 mm) and coupled mechanism draining (D = 10 mm and 12.5 mm). In (**a**,**b**), curves and points in same color depict the respective theoretical and experimental draining profiles for the same input conditions.

Further comparison of model prediction with experimental data are shown in Fig. 7. The figure compares the draining profiles as obtained from theory with experimental data denoted as points in Fig. 7(a,b). The theoretical profile and experimental data for the same input conditions are depicted with the same color to facilitate comparison. We note better predictions of draining profiles for smaller tube diameters and lower surface tension. Nevertheless, the predicted time of draining is more or less same from experiment and theory. This is further evident from Fig. 7(c) which compares the theoretical and experimental data on *T*_*D*_ over the entire range of input conditions. The figure displays satisfactory agreement within ±10% for most of the cases although for higher *D* (lower *Eo*), a higher deviation upto 20% is noted when coupled mechanism draining (full bore and film draining) occurs. A closer observation reveals that the theory slightly under-predicts the time of draining for lower conduit diameters (D < 6 mm), whereas for larger diameter tubes the trend is reverse. It is felt that surface tension induced instability not considered in the analysis would ensure more accurate predictions.

## Effect of Inclination

**Figure 8:**
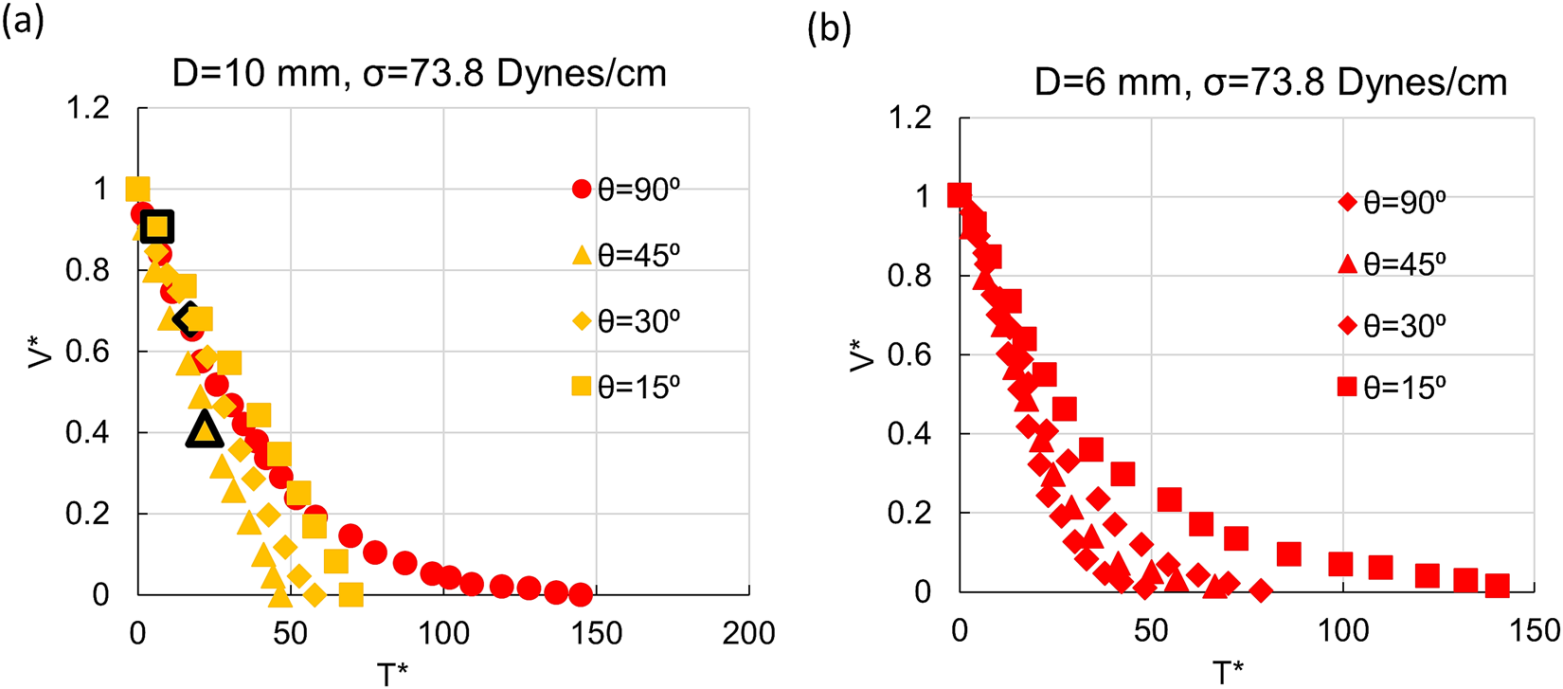
Effect of conduit tilt on draining profile for (**a**) D = 10 mm, β = 0.039 (**b**) D = 6 mm, β = 0.065. Black markers denote Taylor finger entry.

The draining phenomena becomes more fascinating in inclined conduits. Since liquid drains along the lower side of the inclined tube and facilitates air entry through the crescent shaped passage between the draining film and the conduit wall, there is a greater propensity of finger entry with increase in inclination for same Eo and β (Fig. (8a)). This results in an increasing range of film and coupled mechanism draining accompanied by the disappearance of Galilean regime with increase of θ. This is evident from the regime maps presented in Fig. 5(b,c).

The evolution of draining time with tilt is also different with and without finger entry (Fig. 8). As expected, tubes drain faster when inclined more under full bore draining conditions (Fig. 8(b)). However, with finger entry, the rate of draining increases till a critical conduit inclination and henceforth decreases (Fig. 8(a)). This can be corroborated with the behavior of Taylor finger in inclined conduits^6^ and further suggests that draining in tilted conduits is predominantly influenced by the dynamics of Taylor finger growth. This also suggests that complete emptying especially in inclined conduits occurs only when a Taylor finger enters the conduit.

## Conclusion

The paper discusses experimental investigations and theoretical analysis to understand the complex physics of draining involved in an inverted Clepsydrae geometry. The phenomena, not explored till date, is compared with closed top and open top draining, well documented in literature. Based on experimental observations, we propose a phase diagram displaying film draining, full bore draining and the transition where both mechanisms prevail. We have also attempted to quantify the “smallness” of top pierce where only film draining prevails and its “largeness” which marks the inception of Galilean draining. We further note that draining from closed top tubes can be significantly facilitated by a minute pierce of the top closure or by a mere tilt of the conduit which enhances the tendency of Taylor finger inception. The mechanistic model validated with experiments can predict the time of draining with and without finger entry within ±10% in the meso-scale.

## References

[1] Clanet C (2000). Clepsydrae, from Galilei to Torricelli. Phys. Fluids.

[2] Davies RM, Taylor G (1950). The Mechanics of Large Bubbles Rising through Extended Liquids and through Liquids in Tubes. Proc. R. Soc. A Math. Phys. Eng. Sci..

[3] Fabre J, Liné A (1992). Modeling of two-phase slug flow. Annu. Rev. Fluid Mech..

[4] Clanet C, Searby G (2004). On the glug-glug of ideal bottles. J. Fluid Mech..

[5] Kannan A, Ray S, Gargi D (2015). Liquid-liquid flow patterns in reduced dimension based on energy minimization approach. AIChE J..

[6] Zukoski EE (1966). Influence of viscosity, surface tension, and inclination angle on motion of long bubbles in closed tubes. J. Fluid Mech..

[7] Colebrook CF (1939). Turbulent Flow in Pipes, with particular reference to the Transition Region between the Smooth and Rough Pipe Laws. J. Inst. Civ. Eng..

[8] Bird, R. B., Stewart, W. E. & Lightfoot, E. N. *Fluid mechanics with engineering applications*. (John Wiley & Sons, 1960).

[9] Daugherty, R. L. *Fluid mechanics with engineering applications*. (Tata McGraw-Hill Education, 1989).

[10] Jana AK, Mandal TK, Chakrabarti DP, Das G, Das PK (2007). An optical probe for liquid-liquid two-phase flows. Meas. Sci. Technol..

[11] Wallis, G. B. One-dimensional two-phase flow, McGraw‐Hill, New York (1969)

[12] Bretherton F. P. (1961). The motion of long bubbles in tubes. Journal of Fluid Mechanics.

[13] Taylor G. I. (1961). Deposition of a viscous fluid on the wall of a tube. Journal of Fluid Mechanics.

[14] Irandoust S, Andersson B (1989). Liquid Film in Taylor Flow through a Capillary. Ind. Eng. Chem. Res..

